# Transferable approaches to CRISPR-Cas9 induced genome editing in non-model insects: a brief guide

**DOI:** 10.1186/s12983-025-00566-2

**Published:** 2025-07-07

**Authors:** Hassan M. M. Ahmed, Lisha Zheng, Vera S. Hunnekuhl

**Affiliations:** 1 Department of Developmental Biology, Johann-Friedrich-Blumenbach Institute, GZMB, University of Göttingen, Göttingen, Germany; 2 Department of Crop Protection, Faculty of Agriculture – University of Khartoum, 13314 Khartoum North, Sudan; 3 Department of Evolutionary Developmental Genetics, Johann-Friedrich-Blumenbach Institute, GZMB, University of Göttingen, Göttingen, Germany

**Keywords:** CRISPR-Cas9, Genome editing, New model organisms, Insects

## Abstract

Despite the large variety of insect species with divergent morphological, developmental and physiological features questions on gene function could for a long time only be addressed in few model species. The adaption of the bacterial CRISPR-Cas system for genome editing in eukaryotic cells widened the scope of the field of functional genetics: for the first time the creation of heritable genetic changes had become possible in a very broad range of organisms. Since then, targeted genome editing using the CRISPR-Cas technology has greatly increased the possibilities for genetic manipulation in non-model insects where molecular genetic tools were little established. The technology allows for site-specific mutagenesis and germline transformation. Importantly, it can be used for the generation of gene knock-outs, and for the knock-in of transgenes and generation of gene-reporter fusions. CRISPR-Cas induced genome editing can thus be applied to address questions in basic research in various insect species and other study organisms. Notably, it can also be used in applied insect biotechnology to design new pest and vector control strategies such as gene drives and precision guided Sterile Insect Technique. However, establishing CRISPR in a new model requires several practical considerations that depend on the scientific questions and on the characteristics of the respective study organism. Therefore, this review is intended to give a literature overview on different CRISPR-Cas9 based methods that have already been established in diverse insects. After discussing some required pre-conditions of the study organism, we provide a guide through experimental considerations when planning to conduct CRISPR-Cas9 genome editing, such as the design and delivery of guide RNAs, and of Cas9 endonuclease. We discuss the use of different repair mechanisms including homology directed repair (HDR) for a defined insertion of genetic elements. Furthermore, we describe different molecular methods for genetic screening and the use of visible markers. We focus our review on experimental work in insects, but due to the ubiquitous functionality of the CRISPR-Cas system many considerations are transferable to other non-model organisms.

## Introduction

### CRISPR-Cas9 genome editing: new horizons for non-traditional models

The bacterial repetitive sequence known now as CRISPR (clustered regularly interspaced palindromic repeats) was observed for the first time in the late 1980s [1–3]. In the following, the ground-breaking discovery that this system could be highjacked for targeted genome editing in eukaryotic cells [4] paved the way for its use in cultured cells, in model organisms and for medical applications [5–7]. The creation of a DNA double strand break (DSB) also greatly increases the efficiency of foreign DNA insertion into a locus, making CRISPR a prime tool for targeted transgenesis [8–10]. Researchers working in established genetic systems quickly adapted CRISPR-Cas for their model, also using it for more sophisticated applications like the precise editing of a locus [11, 12], the creation of genetic knock-ins [13–15] and for driving CRISPR guide RNAs and Cas9 in a tissue specific fashion [16–18]. But notably, the CRISPR-Cas9 technique also held great promise for the community of researchers working on molecular genetic questions in non-traditional models, some of which resistant to RNA interference, until then the only method available for reverse genetic targeting to study gene function [19]. CRISPR-Cas9 is believed to work on every animal genome where guide RNAs and Cas9 protein can be delivered into a cell. Wherever germ cells can be targeted, and the study species can be bred in the lab, the generation of stable genome edited lines has become possible.

Despite these promises, the application of CRISPR-Cas9 genome editing in a new organism can be complicated by technical issues such as form and delivery of the components, design of suitable guides with low risk of off-target cuts, and the identification and assessment of successful editing events. This brief guide is intended to lead through the literature on the use of CRISPR-Cas9 in non-model insect species. We review different strategies for mutagenesis and transgene knock-in that can be chosen based on the experimental question and the study organism. We also outline how to practically apply CRISPR-Cas9 to a new organism by describing guide RNA (gRNA) design, production and efficiency testing as well as different strategies for gRNA, Cas9 and repair template delivery. Finally, we discuss methods for molecular screening and stock building. Although we focus our review on insects, most considerations are transferrable to other organisms due to the generic functionality of CRISPR-Cas9 in eukaryotic cells.

### CRISPR-based techniques have been established in various insects

**Fig. 1:**
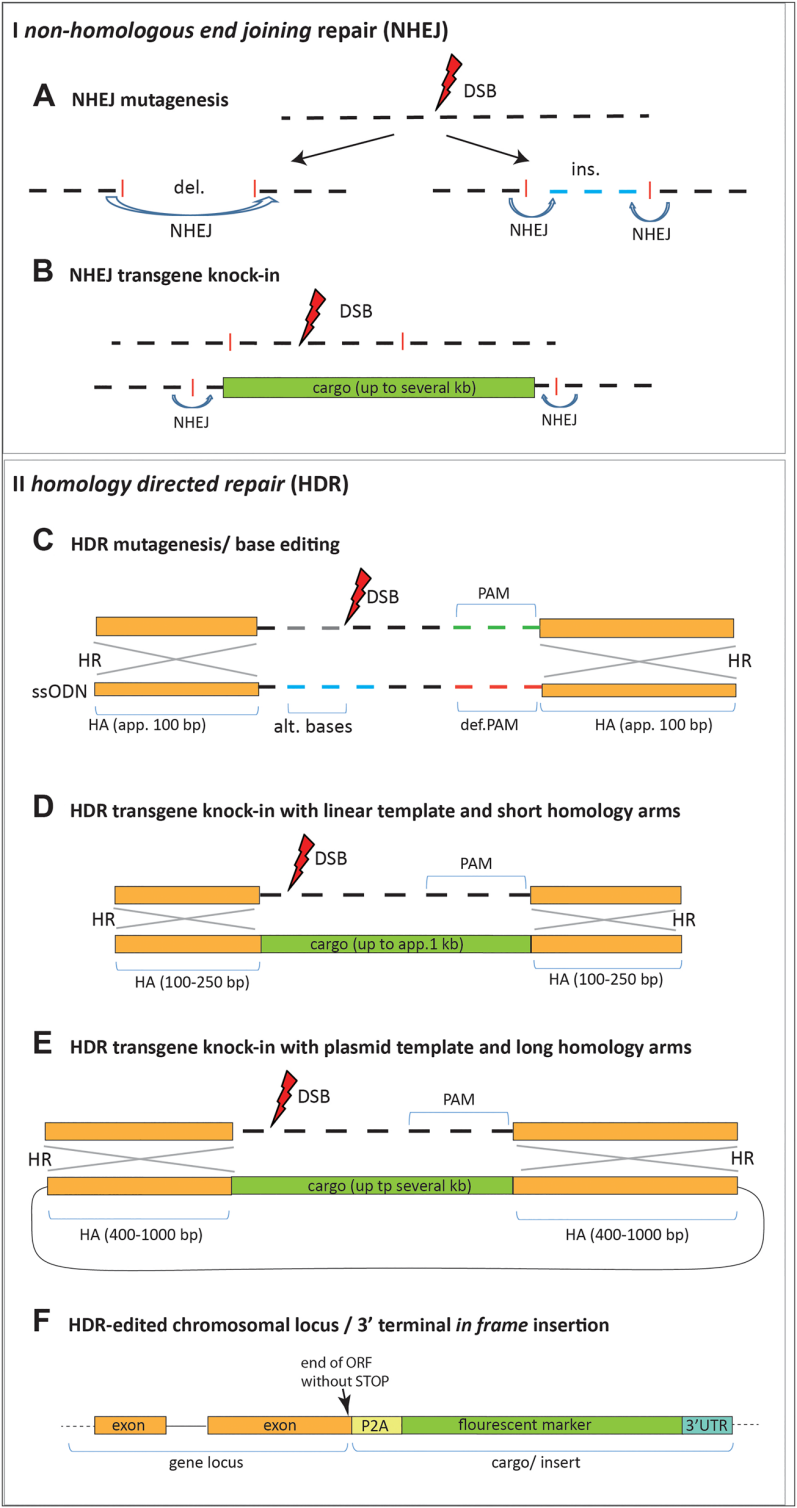
Strategies for CRISPR-Cas9 induced mutagenesis and transgenesis using the non-homologous end joining repair (NHEJ, I) and homology directed repair (HDR, II). **A** CRISPR-Cas9 induced double strand break (DBS) is repaired by the ligase IV mediated NHEJ mechanism, often leaving small deletions or insertions and by these means mutating the gene. **B** If a linearized repair template is provided it can be integrated into the genome by NHEJ at random orientation. **C** Precise, small scale base editing can be achieved by inducing a DSB in the target site and providing a short single stranded oligodeoxynucleotide (ssODN) in which a short insert in flanked by homology regions of app. 100 bp per side to induce homologous recombination (HR). The insert alters individual bases to create a defined mutation and can also alter the PAM to avoid re-mutagenesis of the edited locus. **D** Linearized repair templates with short homology regions from 40 bp per side have successfully been used to integrate cargoes of up to 1 kb upon CRISPR-Cas9 DSB induction. **E** Large cargoes of several kb have been integrated into the genome under the use of HDR repair plasmids on which the transgene is flanked by homology arms of 400–1000 bp. Again, the PAM of the CRISPR target site should be omitted from the homology arm if possible. If PAM containing sequence is needed for in frame translation the motif should be altered (to synonymous base triplets) in the homology arm region of the repair plasmid. **F** Insertion of a fluorescent marker protein at the 3’end of a gene, including a 3’ untranslated region (UTR) with a polyadenylation site. In frame insertion by HDR leads to simultaneous transcription with the gene in one open reading frame (ORF), but the proteins are separated by the 2A peptide (P2A). Abbreviations: alt. = alternative, def. = defective

The simplest application of the CRISPR-Cas9 system is creating mutant alleles of coding genes in which CRISPR-Cas9 induced *double strand breaks* (DSBs) are repaired by erroneous *non-homologous end joining* that leaves small insertions or deletions (indels) disrupting the reading frame (Fig. 1A). Most insects that have been injected with mutagenic CRISPR reagents show a mosaic phenotype in which a proportion of cells carry the mutant allele, and others remain unaltered. Phenotypic analysis of the treated/injected generation (G_0_-animals) that takes this mosaicism into account is possible even in species where germline targeting and isolating mutant alleles may prove difficult. As a test of functionality, visible markers, as for example eye pigmentation genes, were often targeted in the first instance for establishing the technique and G_0_ injected animals displayed varied degrees of mosaicism [20–24]. In some species it is also possible to target the embryo at the one-cell stage so that some G_0_ individuals display the full homozygous mutant phenotype [25–27]. An elegant example of G_0_ mutant analysis comes from the crustacean *Parhyale*. Due to the fixed cell lineage in which left and right side of the animal go back to the blastomeres of the 2-cell stage, injecting one of these blastomeres led to gene knockout on one side of the body while the other half remained wild type [28].

To create heritable genomic changes the CRISPR-Cas9 components must be delivered to the germline cells. G_0_ injected animals are then backcrossed to wildtype individuals. The F_1_ generation (offspring of G_0_founders crossed to wildtype animals) is screened for desired mutations using different strategies from visible phenotypes such as eye or cuticle pigmentation to molecular approaches including PCR-based genotyping. Inbreeding of mutant allele carriers allows for the creation of homozygous mutants that display a full loss-of-function phenotype. As for mutagenesis and G_0_-analysis, germline transformation has been successfully applied in various insects, including holometabolan species [10, 29], hemimetabolan species [20, 30, 31] and in Zygentoma, a basal branch of ametabolous insects [32].

CRISPR-Cas9 cannot only be used for the creation of mutants but also for introduction of transgenes into the genome as the induction of DSBs greatly increases the efficiency of integration/knock-in of provided foreign DNA fragments into a locus [33, 34]. Since DNA DSBs are toxic for the cells, several mechanisms have evolved to promptly repair them. Among those the *non-homologous end joining* (NHEJ) (Fig. 1A, B) and the *homology directed repair* (HDR) (Fig. 1C–F) are mainly exploited to knock-in foreign DNA (see [35–37]).

NHEJ based knock-ins (Fig. 1B) work quite efficiently in the zebrafish [9, 14, 15] and among insects, knock-ins using NHEJ-repair mechanism have been achieved in the beetle *Tribolium castaneum* [38] as well as in the orthopterans *Acheta domesticus* and *Gryllus bimaculatus* [39, 40]. However, as this repair mechanism does not rely on homology between the provided repair template and the genomic target, it is imprecise and does not allow for the base-by-base editing which is required for creating fusion proteins or bicistronic reading frames. The most elegant and faithful way of integrating fragments is to make use of the cellular HDR mechanism (Fig. 1C–F). Provision of homology arms flanking the intended insert allows to precisely define the sequence of the targeted allele [12, 36, 37]. HDR can be utilized to knock-in large DNA fragments. This has been successfully demonstrated not only in *Drosophila* species [8, 12, 41, 42], but also in some other more developed genetic models such as the beetle *Tribolium castaneum* [43], in some mosquito species [44–48] and in hymenopterans [49]. It is also possible to use CRISPR-Cas9 induced HDR for short insertion or to create defined mutations [10, 22].

### Use of CRISPR-Cas9 for the generation of gene drives and other methods of insect pest and vector control

Not only has CRISPR-Cas9 been adopted to answer questions in basic research but also in applied insect biotechnology that aims to develop transgene-based pest and vector control strategies. It has been intensively used to engineer novel gene drive systems in which a cassette including Cas9 and a gRNA was inserted within its target gene so that when it cuts the homologous chromosome the cassette is copied to it, resulting in super-mendelian inheritance of the cassette. Such gene drives can be tailored to achieve insect population suppression [46, 50] or modification [51]. The CRISPR-Cas9 system has also been used to engineer sex ratio distortion to produce more males in order to reduce the targeted insect population [52]. Likewise, the CRISPR-Cas9 system has also been used to improve the sterile insect technique (SIT) to produce competent sterile males for field release. In precision guided SIT (pgSIT) Cas9 endonuclease is used to knockout male-specific genes that are expressed during spermatogenesis leading to male sterility manifested as embryonic development arrest. Ideally, mutant alleles of such genes have no effect on the males’ nor on the sperm’s fitness. Techniques for pgSIT have been developed in flies [53] and in disease vector mosquito species [54, 55] as well as in the invasive moth *Cydia pomonella* [56]. As an alternative for ionizing irradiation CRISPR-Cas9 has also been proposed to be used for the production of sterile males through induction of many simultaneous DNA double strand breaks. Upon release these sterile males are expected to outnumber males of the natural population and by this means suppress population growth [57]. As an alternative strategy to suppress populations of the malaria vector *Anopheles gambiae*, CRISPR-Cas9 pgSIT has been used to confer a female-lethal phenotype [58] or to target female fertility [46].

## Pre-conditions to perform CRISPR experiments

### Preconditions for CRISPR mutagenesis

There are two major prerequisites to establish CRISPR-Cas9-based mutagenesis in a new organism. First, the availability of the genome sequence of the target organism, or at least the sequence of the target gene, to be able to design suitable gRNAs. It is however highly recommended to work with a whole genome sequence to be able to perform an off-target analysis on the gRNA sequence (see section Single guide RNA design and off-target prediction). It is also of paramount importance to re-sequence the target region from the strain that is going to be used to exclude strain-specific polymorphisms in the gRNA recognition sequence [37]. Second pre-condition is the accessibility of the eggs and the possibility to perform embryonic microinjections. Note that this is actually one of the main hurdles for the application of genome editing: hundreds of embryos will have to be injected at a very early stage to have a realistic chance of successful genome editing. To circumvent this requirement it may in some species also be possible to inject gravid females with gRNAs and Cas9 in the form of a ribonucleoprotein complex which is taken up into the oocytes during the vitellogenic phase ([22, 59]/see section Cas9 and sgRNA delivery by ReMot and DiPa CRISPR).

### Preconditions for germline modification

For modification of the germ line the same conditions as above need to be given. In addition, it is advantageous if the generation time of the species is not too long, and it must be possible to perform defined crosses with the injected animals. There is also a necessity to either use a visible marker based on which mutagenized/transformed animals can be identified, or to be able to perform genotyping without sacrificing the animal (see section Editing the germline and establishment of a mutant stock). Alternatively, it is also possible to produce G_0_ individuals with biallelic knockout and without mosaicism for direct analysis (Fig. 4B) [25–27]. In honeybees, it was possible to obtain 59% of G_0_ individuals with biallelic stop/stop for the sex determination gene *doublesex* [25]. This requires injection at the embryonic one cell stage which is not possible in all insects.

## Guide RNAs

Guide RNAs (gRNAs) are crucial for targeting Cas9 endonuclease to the genomic site to be edited. The term guide RNA refers to any RNA molecule that directs Cas9 to a genomic target. Whereas bacterial guide RNAs have a dual structure, single molecule gRNAs have been engineered (see below) for the use in eukaryotic genome editing [4].

### Chimeric single guide RNAs (sgRNAs)

The bacterial CRISPR locus encodes CRISPR RNA (crRNA) in the form of an array in which variable, often virus-derived RNA coding stretches are interspaced with palindromic repeats and trans-acting crRNA (tracrRNA) [4, 60]. The variable part is also referred to as spacer and its genomic origin (protospacer) is upstream of a 3-nucleotide motif called protospacer adjacent motif (PAM). The protospacer is practically the genomic target and the PAM is required for unwinding of the double strand DNA. In the bacterial immune defence tracrRNA and crRNA interact with each other through complimentary base pairing and then form a complex with Cas9 protein. The variable part of the crRNA sequence (guide region) directs Cas9 to the target sequence and invading genetic elements are cut, usually 3 nt upstream of the PAM sequence [4].

**Fig. 2:**
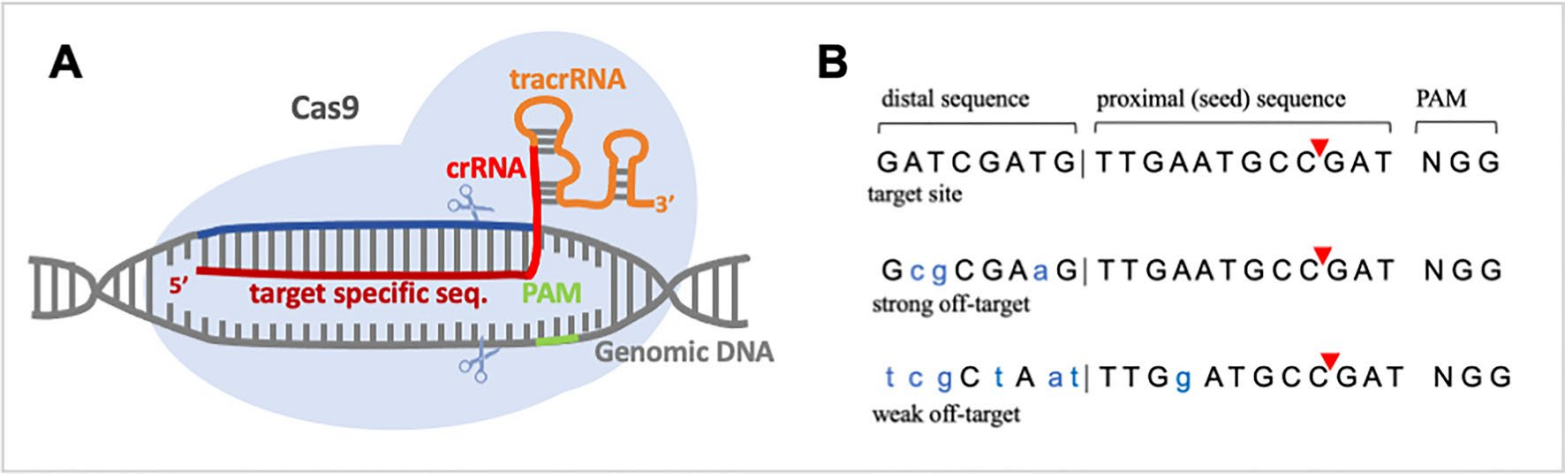
Single guide RNA structure and target site selection. **A** Structure of an sgRNA comprising a crRNA including the guide region, and tracrRNA. SgRNA form complexes with Cas9 protein and guide the endonuclease to their genomic recognition motif (blue). The double strand break is created 3 bp upstream of the genomic PAM (NGG) motif (indicated by scissors). Based on [4, 36]. **B** Top row: sequence of an exemplary sgRNA genomic binding sequence including proximal sequence, distal seed sequence, site of double strand break induction (red arrowheads) and PAM. Middle row: potential strong off-target sequence with few mismatches in the distal sequence. Bottom row: weak off-target sequence with mismatches in distal and proximal sequence. Based on CRISPR Optimal Target Finder at http://targetfinder.flycrispr.neuro.brown.edu/. Classifications given here only reflect tendencies, full criteria can be found in [41]

To facilitate their use in genome editing, tracrRNA and crRNA have been engineered into a continuous chimeric single guide RNA (sgRNA) with the customizable target-specific 18–20 bp sequence at the 5’ end (guide region) [4, 61] (see Fig. 2A).

### Single guide RNA design and off-target prediction

Guide regions of sgRNAs can be designed using available online bioinformatics tools such as *CRISPR Optimal Target Finder* at http://targetfinder.flycrispr.neuro.brown.edu/ [41] (and alternative tools are available at https://chopchop.cbu.uib.no/ [62] or https://crispr.dbcls.jp/ [63]). After providing the target sequence these tools will return possible sgRNA target sites that are adjacent to a PAM (*NGG*) ranked by specificity. If the genomic sequence of the organism has been included in their database these tools can also be used for an off-target analysis. If not, potential off-targets can be identified by searching the genome using the suggested guide sequence including the PAM as a query. The 12 nucleotides of the guide region that are directly adjacent to the PAM are the seed sequence (or proximal sequence), followed by an 8 nt long distal sequence (see Fig. 2B). Multiple mismatches can be tolerated in the distal sequence whereas the seed sequence is less tolerant to mismatches: it is normally required as an exact match or with only 1 bp mismatch for a sequence to pose an off-target risk. Comprehensive criteria for off-target evaluation used by *CRISPR Optimal Target Finder* are given in [41]. Wherever possible we recommend using sgRNAs without any predicted off-targets.

### Testing sgRNAs

Not all potential sgRNAs have the same efficiency and therefore it is advisable to test the activity of several sgRNAs before indulging in laborious experiments. We recommend in vivo testing of sgRNAs as the efficiency might be influenced by aspects such as chromatin accessibility that would not be reflected in in vitro testing [64–66]. For in vivo testing embryonic injections of the sgRNAs along with Cas9 protein or plasmid are performed (see below). After some maturation time the developed injected embryos or first instar larval hatchlings are used for genomic DNA extraction. The molecular testing of guide efficiency can then be done by using one of the methods that are described in section Assessment of mutation rates. Additionally, it is recommended to test guide RNA efficiency at different concentrations, and at different concentrations of Cas9 protein/plasmid (see below), to define the optimal experimental parameters for high editing rates. It has to be noted that guide RNA testing is by itself a laborious experiment. Alternatively, several guide RNAs can be applied in parallel experiments and the setup with the best editing results can be chosen for further analysis.

## Form and production of CRISPR-Cas9 reagents

**Fig. 3:**
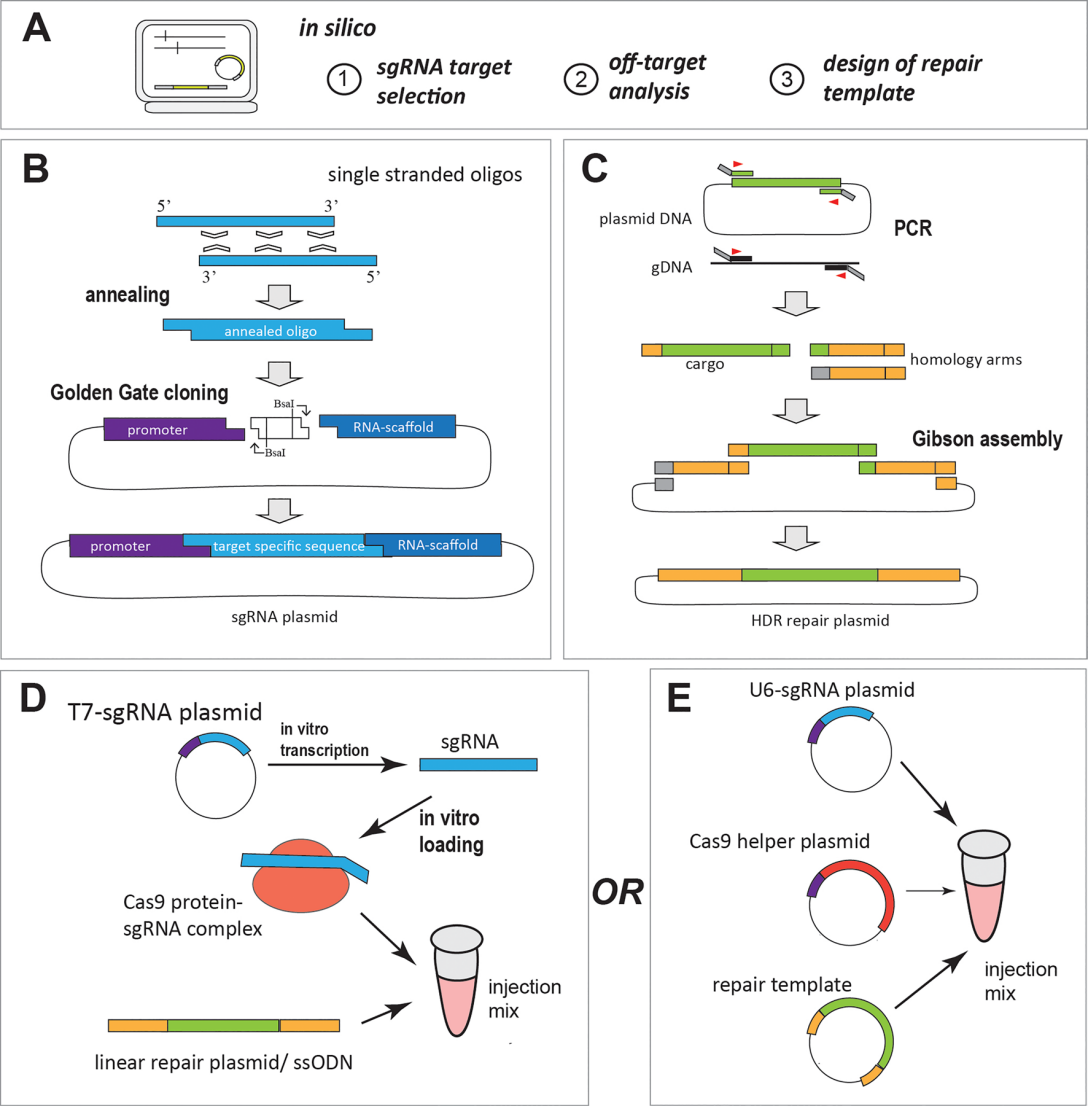
Workflow for preparing CRISPR-Cas9 genome editing. **A** In silico preparations: 1) design of guide region of sgRNA. Different tools are available for the selection of suitable target sequences for off-target analysis, and design of the repair template. Guides can be ordered as oligos with a 3’ sequence suited for cloning into a respective vector. **B** Cloning of the variable part of a sgRNAs. Forward and reverse oligos encoding the guide region are annealed creating overhangs required for cloning. The annealed double stranded oligo is cloned into a vector that provides the required RNA promoter as well as sequence encoding the invariant part of crRNA and tracrRNA (RNA-scaffold). Golden Gate cloning using *Bsa*I (which creates a staggered cut outside its recognition motif) can be used [37, 43]. **C** A seamless repair plasmid suitable for HDR can be produced using Gibson assembly. For this the cargo sequence is amplified using overhang primers that create 5’ and 3’ sequences matching the respective ends of the homology arm sequence, which are amplified from genomic DNA and carry sequences matching the end of the cargo sequence. All fragments are fused by the Gibson assembly exonuclease digest that creates complementary overhangs by digesting one strand starting from 5’ [69]. **D** SgRNAs can be transcribed in vitro under the use of T7 RNA polymerase and the respective promoter. SgRNAs are then loaded on Cas9 ribonucleoprotein. A repair template can be provided in a linearized or circular form and is added to the injection mix together with the Cas9-sgRNA complex. If desired the 5–10% of the vital dye phenol red can be added to the injection mix to increase visibility of the injection process. **E** Alternatively, sgRNAs can be provided on plasmids under the control of an endogenous RNA promoter (e.g. U6) [43], along with a plasmid from which Cas9 is transcribed from a ubiquitous promoter, and a repair plasmid. In vivo transcribed sgRNAs can also be combined with Cas9 protein. Again, phenol red can be added to the mix if desired

The workflow of preparing an injection mix that includes all necessary reagents for a CRISPR genome editing experiment is summarized in Fig. 3A–E.

### Preparation of sgRNAs

Once suitable guide regions with no or minimal genomic off-targets are identified (see section Guide RNAs, Figs. 2B, 3A), they can be directly ordered from some companies as ready to use sgRNAs. Alternatively, they can be in vitro transcribed using a DNA template in which the sgRNA coding sequence is fused to a viral RNA polymerase promoter such as T7. For efficient transcription using T7 RNA polymerase, the first two nucleotides of the sgRNA should be GG.

The most cost-effective form of delivery of sgRNAs is in form of plasmid DNA from which the sgRNA is transcribed by an *RNA pol III* promoter such as the promoter of the *U6 small nuclear RNA* gene [67]. To deliver sgRNAs in this form, it is recommended to use endogenous promoters as there seems to be limited cross species functionality of core promoter sequences [42, 43, 68]. To achieve optimal transcription of sgRNAs from the commonly used *U6 RNA pol III* promoter the first nucleotide to be transcribed must be a G. If the guide sequence starts with any other nucleotide, this should be replaced by a G. CRISPR Optimal Target Finder tool allows for selecting both, G (for U6-promoter) or GG (for T7-promoter), at the 5’ end of the customizable guide sequence [41]. Several vectors with suitable promoters, sgRNA scaffold and transcription termination signal have been designed by different groups (e.g. [43]) and have been made available through the nonprofit repository addgene (https://www.addgene.org/). These vectors are designed with two recognition sites for outside cutter type II restriction endonucleases such as *Bsa*I and *Bbs*I to facilitate seamless cloning of the variable part of the sgRNA between the RNA pol III promoter and the sgRNA scaffold using the golden gate strategy. To insert the variable part of the sgRNAs into one of these vectors (e.g. https://www.addgene.org/65956/) two complementary single stranded DNA oligoes specific to the target site with additional 4 nucleotides at their 5’ ends that are compatible to the overhangs generated when the vector is digested by the respective outside cutter are annealed to create a double stranded oligo with overhangs compatible for Golden Gate cloning [37, 43] (see Fig. 3B).

#### Co-application and multiplexing of sgRNAs

A general anticipation is that designing and co-applying multiple sgRNAs to one target gene will improve the knockout efficiency. Multiple guides can be produced in vitro and co-injected. Alternatively, if sgRNAs are supplied on a plasmid for in vivo transcription, multiple plasmids that carry a single sgRNA can be co-injected, or one plasmid can contain multiple sgRNAs. in the latter case, sgRNAs can be driven by individual promoters, although it is recommended to use different promoters for each sgRNA to avoid recombination between identical promoter sequences [70]. Another strategy for multiplexing sgRNAs exploits the highly conserved processing system of the transfer RNAs (tRNAs) by ribonuclease P and Z into individual functional tRNAs as they are naturally produced as long transcripts carrying several copies of the respective tRNA [71, 72]. When using this system for multiplexing, several sgRNAs flanked by tRNA encoding sequence are driven by a single promoter producing a single transcript. The ribonucleases P and Z recognize and process the transcript into individual tRNAs which also leads to release of individual functional sgRNAs [18, 70, 73]. It has been observed that two sgRNAs in a multiplexing system are highly efficient whereas the inclusion of additional sgRNAs may not always lead to a further increase in mutation rate [16, 18, 74]. We recommend using species specific tRNA sequences if possible, although tRNA sequences do also show cross-species functionality [18].

### Molecular form of Cas9

Cas9 can be delivered in the form of plasmid in which the coding sequence (ideally insect codon optimized) is fused to a nuclear localization signal (NLS) and is cloned behind a constitutive (e.g. *Act5c*, *pUb* [75–77]), inducible (*hsp70* [78]) or germline-specific (e.g. *vasa* [79]) enhancer/promoter. Recombinant Cas9 protein including a NLS can also be produced in bacterial cells and purified in the lab or purchased from different suppliers. In this case Cas9 protein is mixed with synthetic or in vitro transcribed sgRNAs to form the functional ribonucleoprotein (RNP) complex before delivery (see Fig. 3D) [42]. Whereas plasmid injection (Fig. 3E) might be more convenient in species for which functional promoters are available, the use of Cas9 protein in injection mixes is more versatile and has proven to work efficiently (e.g. [21]). This method can also be used in ReMot and DiPa-CRISPR experiments (see section Cas9 and sgRNA delivery by ReMot and DiPa CRISPR/[22, 59]). Cas9 can also be provided as mRNA [80], but single stranded RNA generally is less stable than protein or plasmid DNA.

#### Alternative Cas proteins

Whereas Cas9 is the most widely used CRISPR endonuclease there are other Cas proteins with endonuclease activity but partly divergent features, some of which might be of advantage for some editing approaches [47]. A potentially very useful Cas protein is Cpf1 (Cas12) which uses a T-rich PAM and does not require a tracrRNA but only crRNA. It creates a 5-nt staggered cut. The resulting cleavage product is thought to favour NHEJ based insertion of fragments and designing the ends of the repair templates so that they match these ‘sticky ends’ may allow the precise and oriented insertion of fragments by an HDR-independent mechanism [48]. In transgenic silkworm a Cas12a protein showed a higher efficiency at cleaving viral DNA than a Cas9 protein [50].

## Physical delivery of CRISPR-reagents (sgRNAs and Cas9)

### Embryonic microinjection

**Fig. 4:**
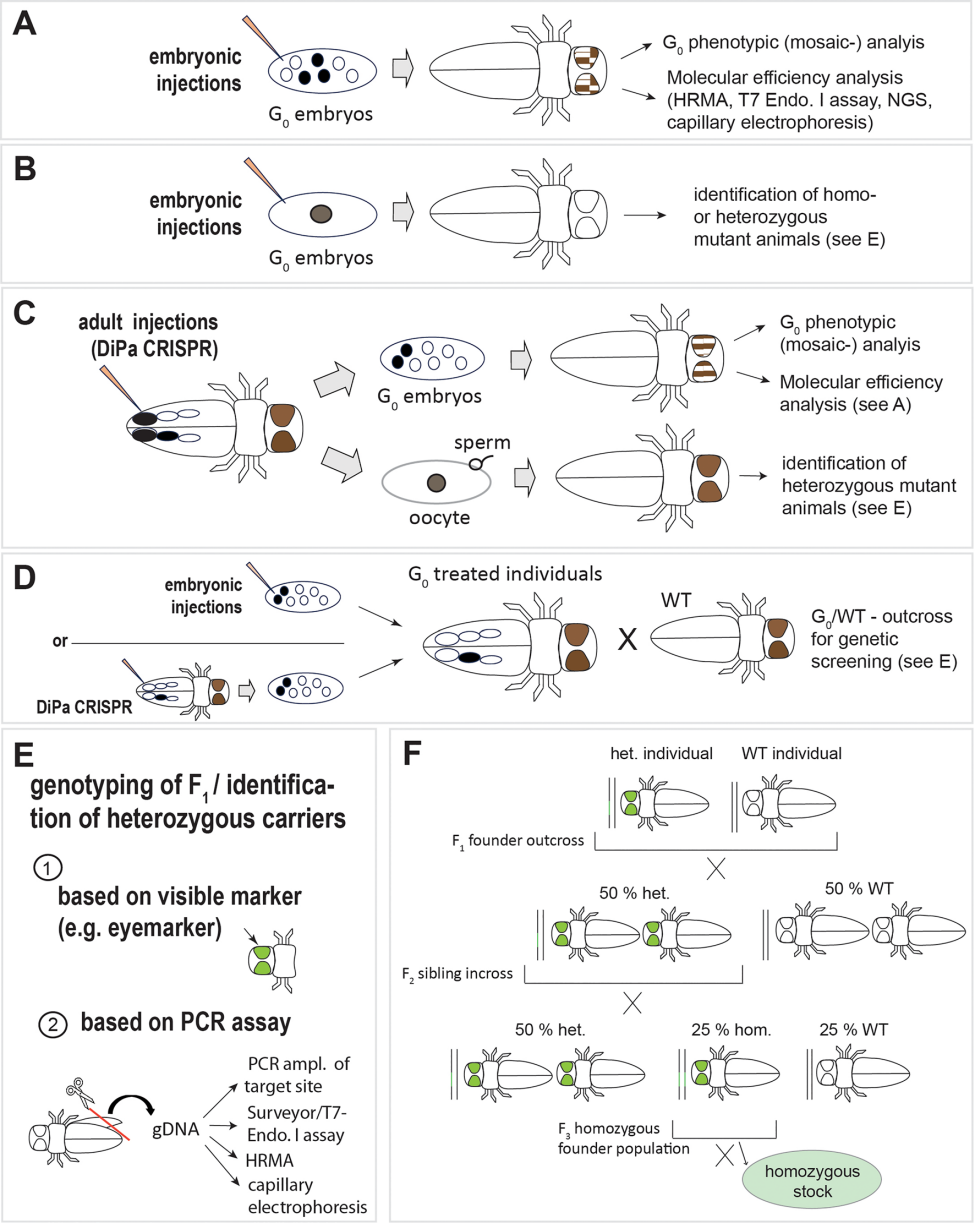
CRISPR delivery, mutant analysis and generation of stable lines workflow. **A** Embryonic injections of CRISPR-Cas9 reagents into the early syncytial stage can directly be followed by a phenotypic analysis. Treated individuals will show varying degrees of mosaicism. **B** Biallelic editing is possible if one-cell stage embryos are injected. Animals with two mutated gene copies display the full homozygous phenotype. **C** Alternative to embryonic injections the haemolymph of gravid females can be injected (DiPa CRISPR or ReMot, see text for required components). A G_0_ phenotypic analysis can be carried out on the offspring of injected females. Similarly, individuals will show varying degrees of mosaicism where the syncytial stage has been targeted (top row). Due to the early application, it is also possible that the oocyte is edited, leading to heterozygous offspring upon fertilization with wild type sperm (bottom row). **D** For germline transformation embryonic or parental injections can be performed depending on the respective study organism. Parental delivery only allows for short repair template (100–200 bp). A subset of G_0_ offspring with mosaicism will carry the mutation or insertion in some germ cells. G_0_ animals are outcrossed to a WT strain to identify those individuals that produce heterozygous carriers of the mutation/ insertion. **E** Heterozygous allele carriers (F_1_) can be identified by either a visible marker (1) or by PCR and duplex assay, HRMA or capillary gel electrophoresis that follows the extraction of genomic DNA without sacrificing the carrier (2). **F** To multiply a transformed allele and to create stable homozygous lines F_1_ carriers are first outcrossed individually to WT individuals. The following generation (F_2_) will consist of 50% heterozygous carriers which are then inbred. This sibling cross produces 25% homozygous animals. Heterozygous carriers of each generation can be identified by a visible marker or by PCR screening. Most markers do not allow distinguishing homo- from heterozygous animals. Therefore, additional molecular analysis or an outbreeding experiment is required for the identification of homozygous animals. These are then inbred to generate a homozygous stock. Abbreviations: het. = heterozygous, hom. = homozygous, WT = wild type

Injections mixes containing sgRNAs together with Cas9, and possibly repair templates (see below, and Fig. 3C–E) are usually injected into eggs of insects as early as possible at the syncytial stage (Fig. 4A) in which the injected material can freely diffuse (e.g. [81]). With every cell cycle the nuclear envelope breaks down and the chromosomal DNA becomes accessible [82]. Early editing events will be transmitted to a larger proportion of daughter cells. To target the germline injections can be directed to the posterior part of the embryo where germ cells specify in some insect species [81]. The mode and location of germ cell formation is however variable among insect species and should be taken into account when planning a germline transformation experiment [83].

### Transgenic Cas9 lines

Most CRISPR genome editing experiments on non-model insects are conducted using Cas9 that is injected along with sgRNAs. Transgenic lines expressing Cas9 endonuclease ubiquitously have been established in order to achieve high editing efficiencies, simplify the injection process and to avoid toxic background effects of Cas9 [84]. It is however in many instances advantageous to express Cas9 endogenously only in the germ cells, e.g. by using germline-specific promoter/enhancer elements. In the model *Drosophila melanogaster* strains that express Cas9 in the germline were generated and are routinely used [8, 84, 85]. As an example, to increase the efficiency of genome editing and to pave the road for Cas9 based pest control strategies of the invasive agricultural pest *Drosophila suzukii*, several transgenic lines that express Cas9 under regulatory elements of *D. melanogaster heat shock protein 70* (*hsp70*) gene [86] and the germline-specific gene *nanos and vasa* were established [53]. Similarly, several germline-specific Cas9 lines of different mosquito species have been established to facilitate genome editing in this insect group that includes disease vectors [51, 87–89]. Outside dipterans we are aware of endogenous Cas9 lines of the fall army worm (lepidoptera) [90] and the beetle *Tribolium castaneum*, although in the beetle endogenously driven Cas9 did not yield a higher genome editing efficiency over injected plasmids [91], which is currently also our experience from working with a different unpublished transgenic *Tribolium* Cas9 line.

### Cas9 and sgRNA delivery by ReMot and DiPa CRISPR

In some insect species the eggs are laid in egg capsules or develop inside the mother and are therefore not accessible for microinjection. Some methods have been developed that allow injection into the haemolymph of egg carrying females and the components enter the oocytes by vitellogenin receptor mediated uptake [22, 59]. Injecting gravid mothers can also be beneficial in species where embryonic injection is possible as it is technically less complex and less time consuming. Another advantage of injecting the mothers is that the oocytes are targeted at a very early stage so that G_0_ animals (eggs laid by the injected females) may show the full heterozygous or hemizygous genotype [29, 59]. One such strategy is called ReMOT an acronym for Receptor-Mediated Ovary Transduction of cargo) in which the Cas9-Ribonucleoprotein complex is fused to a P2C peptide that has been shown to facilitate protein uptake into the ovaries [59]. Subsequently it has been shown that Cas9-Ribonucleoprotein complexes are taken up into the oocytes without being fused to a transport mediating peptide (DiPa-CRISPR = direct parental CRISPR). This method has been quite efficient in *Tribolium* beetles (up to 71% offspring showed somatic mosaicism), and also caused up to 21% mosaic offspring in the cockroach *Blatella germanica* where eggs are not accessible for microinjections [22]. DiPa CRISPR has also been used in combination with short single stranded oligodeoxynucleotides (ssODNs) that also successfully entered *Tribolium* oocytes and introduced small insertions by homology directed repair (see Fig. 1C and section Precise base editing using short repair templates) [22]. However, the uptake of large DNA repair templates (see Figs. 1E, 3C, E) into oocytes embryos by means other than direct injection is to our knowledge currently not possible and the application of ReMot and DiPa CRISPR is therefore restricted to small range edits (see Fig. 1C).

## Using CRISPR-Cas9 to create gene knock-outs

**Fig. 5:**
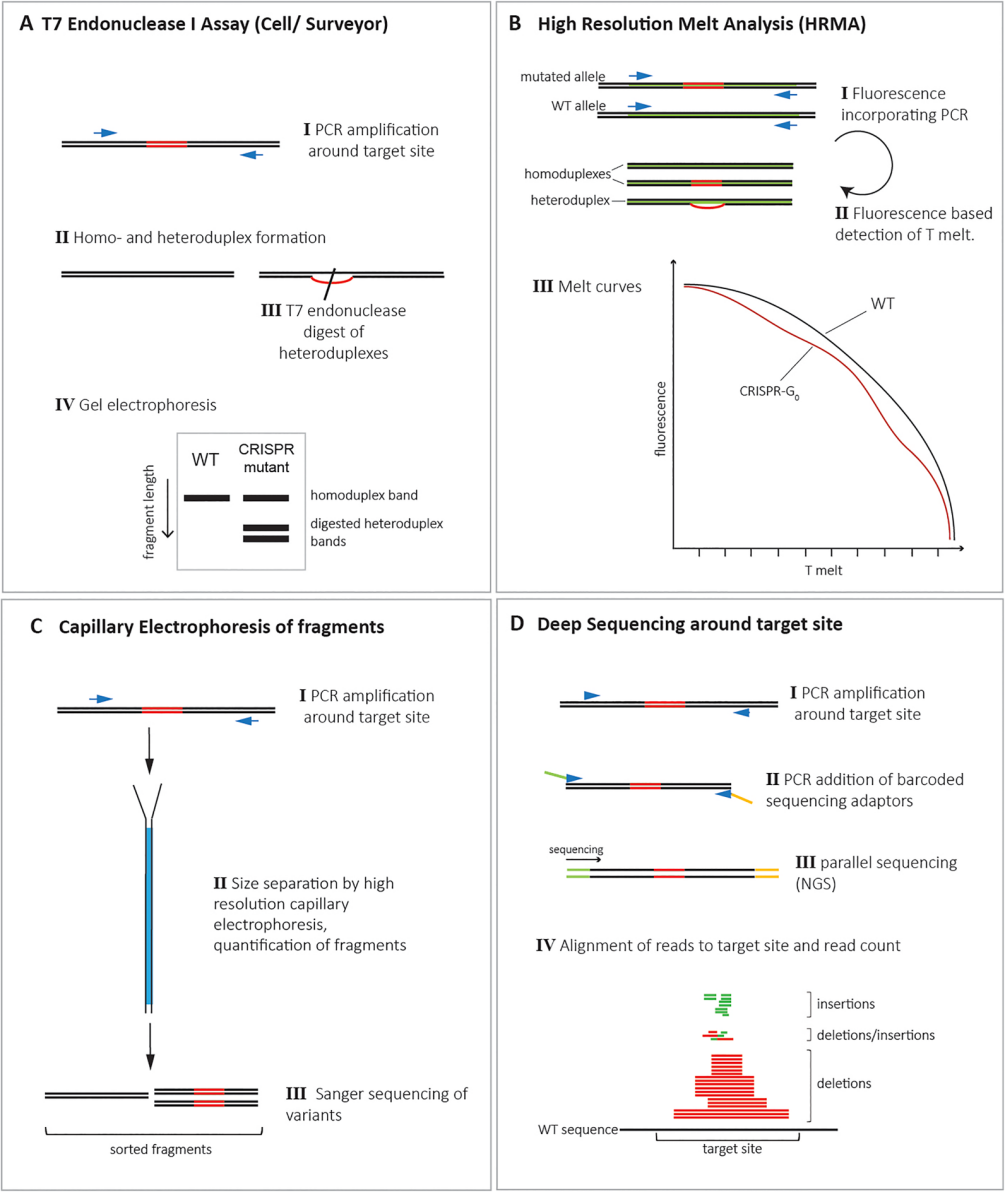
Molecular assays for detection and quantification of CRISPR-Cas9 induced mutations. **A** PCR fragments are designed asymmetrically around the target site. T7 endonuclease-I detects and cuts mismatched DNA. 3 bands will be visible following Gel electrophoresis, representing the longer wildtype sequence and the two unequally sized fragments resulting from the cleavage. Strength of the cleaved bands can give an estimate of editing efficiency. As the assay only detects heteroduplexes resulting from the presence of the wild type allele sample mixing is required for the detection of homozygous mutants. **B** For HRMA primers are designed producing an 80–120 bp fragment including the target site. Under inclusion of a fluorescent dye a quantitative PCR is performed. Heteroduplexes resulting from mutant and wild type allele show lower stability and a lower melt temperature than wild type derived homoduplexes. HRMA can also be used to distinguish wild type, hetero- and homozygous specimens as homoduplexes with an altered sequence will produce a melt curve that differs from the wildtype curve. **C** A 300–500 bp PCR amplicon including the target site is generated and then analysed using high resolution capillary electrophoresis. Small changes in fragment size are detected and the variants are sorted accordingly and quantified. Sorted products are then sequenced. **D** For testing CRISPR-Cas9 guide-RNA efficiency using next generation sequencing (NGS) 100–200 bp around the target site are PCR-amplified. Barcoded sequencing adaptors are added in a second round of PCR and single-end sequencing of the fragments is performed. Alignment of reads to the target sequence identifies the different mutations and read counts allow quantifying the abundance of the individual sequences in the mosaic background. Figure and legend are based on [10, 93, 99, 100, 102]

The major application of the CRISPR-Cas9 system in non-model species is the disruption of gene function allowing for the study of gene function based on the mutant phenotype. In this part we outline different approaches and compare G_0_ mutant analysis (Fig. 4A–C) the generation of stable mutants by germ line transformation (Fig. 4D–F) and characterize the available methods for the molecular assessment of the mutagenized animals (Fig. 5A–C).

### Generation of G_0_ mutants

DNA Double strand breaks (DSBs) that are generated by Cas9 endonuclease are predominantly repaired by ‘non-homologous end joining (NHEJ) (Fig. 1A). This process is error prone and leads to insertions or deletions (indels) of a few nucleotides which in turn result in introduction of premature stop codons or frameshifts within the coding sequence at a high frequency, thus disrupting the reading frame of the target gene [10, 21, 43]. To achieve this, it is important to target the gene close to the translation start codon or at least upstream of important functional domains, to ensure that the introduced mutation leads to complete gene knockout and no partial functional protein is made. G_0_ individuals arising from embryos injected with sgRNAs and Cas9 typically display mosaicism in which the DNA of some somatic cells is mutated while other cells are wildtype-like (Fig. 4A, C). A phenotype can often only be observed in cells where both alleles of the targeted gene have been mutated but a sequence-based assay allows estimating the mutation rate (see below   and Fig. 5). The biallelic mutation rate can be increased by co-injecting multiple guides targeting the same gene or by using a multiplexing system (see section Co-application and multiplexing of sgRNAs) [18, 92]. When injections are performed early it is possible to achieve biallelic editing of the one-cell stage the generation of homozygous mutant G_0_ animals that can be analysed (Fig. 4B) [25–27]. It has been shown that optimizing the concentrations of the CRISPR components and the injection time can lead to more than 50% G_0_ progeny with biallelic knockout of the target gene [25].

### Assessment of mutations rates

Methods that can detect a change in the DNA sequence after CRISPR-based mutagenesis and have successfully been applied for insect genotyping include deep sequencing [10], high resolution melt analysis (HRMA) [93, 94], T7 endonuclease I assay [95, 96] and high resolution capillary electrophoresis [97, 98]. As mutagenesis is commonly applied to interrupt open reading frames and by these means supress protein expression levels it is also possible to use protein detection methods such as western blots. But due to gene self-regulatory effects protein detection methods may produce variable results where a functional wildtype allele is still present, and we do not recommend protein detection as a single method to assess CRISPR efficiency in a mosaic background. The methods described here can also be used for testing of guide RNA efficiency (see section Testing sgRNAs) and the endonuclease assay, HRMA and capillary electrophoresis can also be used for the molecular identification of germ line transformed hetero- and homozygous mutants (see section Editing the germline and establishment of a mutant stock).

#### T7 Endonuclease I / Surveyor nuclease assay

The T7 Endonuclease I and Surveyor nuclease assays are widely used for the purpose of estimating mutagenesis efficiency in a semiquantitative way [99, 100]. They are both based on PCR amplification of around 1 kb fragment from G_0_ injected individuals asymmetrically spanning the target site followed by purification of the amplicon and heating to denature the DNA then allow it to cool down slowly for heteroduplex formation. The T7 Endonuclease I or Surveyor cleavage assay is then performed. Both enzymes recognize the mismatch in the heteroduplex and cleave the mismatched DNA which can then be visualized as three bands: one uncut band and two smaller and unequal bands from the cleavage event (Fig. 5A). The strength of the unequal bands gives an indication on the cleavage efficiency [99]. Note that the bands might be weak and optimization of the experiments may be required. It should also be noted that in case of a sequence polymorphism within the amplicon, as it might often occur in non-isogenic populations, false positive cleavage might be observed. Therefore, it is recommended to sequence the locus before performing the test to identify potential polymorphisms and to carefully evaluate the cleavage products.

#### High resolution melt analysis (HRMA)

HRMA is a PCR-based method for the detection of mutated alleles (Fig. 5B). Primers are designed to generate a 45–150 base pair long, fluorescently labelled amplicon around the CRISPR target site [94]. Where mutations have occurred different homo- and heteroduplexes form. Heteroduplexes are less stable and will melt at a lower temperature compared to homoduplexes generated by amplicons from the wildtype allele, which is reflected in a temperature dependent melting profile generated by a light cycler [93, 101]. HRMA allows for the quantification of mutated sequences in a mixed sample from a mosaic background as the higher the amount of heteroduplex inducing sequences is, the more the melt profile of the sample will differ from the wildtype curve [101]. In addition, sequences with indels have a melting temperature that differs from the wildtype sequence, which allows for the discrimination between wildtype and mutant homoduplexes [101].

#### Capillary electrophoretic separation of DNA fragments

An additional method for automated detection of DNA fragments with CRISPR-induced mutations is the use of a high-resolution capillary electrophoresis system such as QIAxcel Advanced System (Qiagen). PCR amplicons of 300–500 bp around the target site are analysed and fragments that are separated based on a size difference that indicate the presence of indels are further analysed by Sanger sequencing [97] (Fig. 5C).

#### Deep sequencing

The rate at which a targeted genomic site was altered in a CRISPR experiment can also be estimated by a deep sequencing approach of PCR amplicons (Fig. 5D). For this, adaptors that allow for quick library preparation from PCR amplicons are added via overhang primers flanking the target site. It is recommended to keep the rounds of PCR amplification to a minimum to avoid a PCR induced shift in the occurrence of specific amplicons. Next generation parallel sequencing is applied and subsequently sequences are aligned to the genomic site revealing the nature of CRISPR induced indels. Based on this, the proportion of mutated sequence can be estimated [10].

### Precise base editing using short repair templates

CRISPR-Cas9 is often used to create mutations that disrupt gene function by indels due to error-prone NHEJ DSB repair mechanism. The nature of these short indels is however not predictable and will differ between different replicate genome editing experiments. Defined small alterations of the DNA sequence have been successfully achieved in different insect species relying on cellular homology directed repair (HDR) of DSBs using relatively short (100–200 nt) ssODN as a repair template with app. 100 bp long homology arms (Fig. 1C) [8, 10, 22, 103, 104]. Repair template can be designed to introduce a new restriction site in the target sequence that can then be used for the identification of mutants by a combined PCR and restriction digestion assay (RFLP = Restriction fragment-length polymorphism) (see [22, 103]). Alternatively, the Surveyor or T7 Endo I assays can be used to detect allelic mismatches (see 6.2.1, Fig. 5A) [8]. When using ssODNs the sgRNA target sequence should be located as close as possible to the site to be mutated, and the repair template should be designed in a way that it cannot be targeted by the sgRNA (either by changing the proximal sequence of the target, or the PAM sequence or both, see Fig. 1C). This will make the successfully integrated sequence resistant to cleavage by the sgRNA-Cas9 complex. Notably, the use of ssODNs is also compatible with ReMOT and DiPa CRISPR (see section Cas9 and sgRNA delivery by ReMot and DiPa CRISPR) [22, 59].

### Editing the germline and establishment of a mutant stock

Unless editing has occurred in the oocyte or in the zygote (Fig. 4B, C) G_0_ injected animals display cellular mosaicism with respect to the targeted allele (Fig. 4A, C, D). It is only possible for G_0_ individuals to produce offspring carrying the desired gene edits if the genetic change has occurred in the germline (precursor of sperm and oocytes, see Fig. 4D). These G_0_ individuals will produce variable numbers of heterozygous mutated/ transgenic offspring upon outcrossing to wild type specimens (Fig. 4D, F). In many instances, even when pigmentation genes are targeted, heterozygous carriers do not show obvious phenotypes as this would require cells with two mutated alleles. Therefore, heterozygous allele carriers must be identified by genotyping without sacrificing the animal. This can be done by removing a small part (e.g. a leg or a wing), extracting genomic DNA and conducting a PCR based assay (Fig. 4E) [105]. Surveyor/T7 Endo assay, HRMA and capillary electrophoresis are also suitable for identifying heterozygous animals with HRMA being most suitable for high throughput screening (see above, Fig. 5A–C). Given that CRISPR-Cas9 can cause a range of different indels and different germ cells of one individual may carry different mutations, it is advisable to also characterize the mutant allele by PCR and amplicon sequencing and to individually outcross F_1_ carriers to avoid mixing different alleles. Heterozygous F1-founders are then outcrossed to a wildtype animal to obtain a heterozygous F_2_ generation (Fig. 4F).

In cases where the oocyte or the early zygote has been targeted (Fig. 4B, C) resulting homo- or heterozygous individual can directly be used for building up a mutant stock (Fig. 4F) after they have been identified by the same methods described above. It should be noted that homozygous founders resulting from such biallelic editing of the zygote may carry two different mutant allele.

Screening for homozygous animals after the F_2_-siblings have been crossed to one another (Fig. 4F), or after biallelic editing of the zygote (Fig. 4B), is straightforward in case the editing experiment involved targeting of visible markers such as pigmentation genes that have an easy identifiable phenotype where null alleles are present (e.g. *white*, *yellow*, *cinnabar* or *vermillion*) Therefore, these genes are often targeted to establish the CRISPR-technique in a new model [20–22, 106]. However, to answer their actual scientific questions, most researchers aim to target genes of interest whose knockouts will lead to yet unknown, often not instantly visible phenotypes. In these cases, molecular analyses to identify homozygous mutant animals using the same methods as for the identification of heterozygous carriers are necessary (see above).

For many mutant lines homozygous stock keeping may not be possible due to mortality or interrupted fertility of the homozygous animals. In these cases, it is often possible to keep a heterozygous stock instead. For phenotypic analysis two heterozygous animals can then be incrossed and 25% of the offspring will show the full homozygous mutant phenotype.

DNA extraction and molecular screening are laborious, and, in most instances, a high number of potential F_1_-founders must be assessed. A possibility to circumvent this is to include a transformation marker into the mutated site that can be used to identify heterozygous carriers. Insertion of such a marker by *NHEJ*-repair also leads to loss of gene function if the knock-in is disrupting the open reading frame of the gene (see Fig. 1B and section Knock-ins using non-homologous end joining (NHEJ) repair). An eye marker consisting of a fluorescent protein driven by the eye specific 3XP3 enhancer [107] is frequently used for this purpose (Fig. 4 E–F). Alternatively, the ubiquitous ie1 promoter can be used, which offers the advantage that it is expressed broadly at embryonic and larval stages, potentially allowing for earlier identification of transgenic carriers [108].

## Using CRISPR-Cas9 for knock-ins of transgenes

The use of CRISPR-Cas9 also allows for the insertion of foreign DNA, such as conventional reporter genes, into defined genomic location in species that were previously intractable. Here we outline different approaches to CRISPR-based transgenesis. For all knock-in experiments it is advisable to test the sgRNAs (see above) as high mutation rates will greatly improve knock-in efficiencies.

### Knock-ins using *non-homologous end joining* (NHEJ) repair

The NHEJ repair mechanism responsible for fusing DNA ends after a double strand break (and hereby often creating short deletions or insertions, see above) can also be used to knock-in foreign DNA into a locus by providing linear template DNA (see Fig. 1B). This method is not suitable for performing precise genome editing, however, it is a relatively simple strategy to create for example enhancer traps if a reporter gene is placed near the promoter region of a gene [9, 38, 109]. It can also be used for mutagenesis by inserting a visible marker gene into the coding region of a gene, thus interrupting it and allowing for the identification of transformed animals. The efficiency of a NHEJ knock-in may be enhanced by co-injecting multiple sgRNAs targeting the same region [38, 109]. A repair plasmid used in NHEJ must be linearized. The sequence to be inserted using NHEJ strategy has to be linear double stranded DNA, either as a PCR product or linearized plasmid. In case of plasmid, it can either be linearized in vitro by restriction digestion or in vivo by the CRISPR-Cas9 itself. In the latter case, the plasmid should carry at least one non-endogenous CRISPR target site close to the sequence to be inserted and the respective gRNA need to be provided in the injection mix. However, this leads to integration of the whole plasmid including the backbone. Ideally the insert should be flanked at both ends with unique non-endogenous CRISPR target sites and provide the gRNAs. This should lead to generation of two dsDNA molecules; the insert and the plasmid backbone [9]. However, there is theoretically a 50% chance of insertion of the right sequence and 50% for the backbone, plus additional remaining risk of inserting the whole vector if linearization is incomplete. Additionally, regulatory elements of a gene and gene function may be affected by targeting the upstream or intronic region. Therefore, creating knock-ins using NHEJ is often not the method of choice, even though it works very efficiently in some species [9, 38–40, 109].

### Homology directed repair: precise editing with large cargoes using repair plasmids

The cellular homology directed repair pathway (HDR) of DSBs can be exploited for precise genome editing (Fig. 1C–E). For example the gene of interest can be tagged with green fluorescent protein (GFP) in a bicistronic fashion in which the sequence coding for the viral self-cleaving 2A peptide [110, 111] replaces the stop codon and is followed by a sequence encoding GFP that is thus fused to 3’-end of the gene [37, 43]. To achieve the removal of unwanted sequence (in this case the stop codon) guide RNAs are often designed upstream and downstream of the target site to excise the signal. It is however not strictly necessary to have two guides flanking the target as finding guides that do not pose an off-target risk in a short sequence stretch may not always be possible. The sequence of the edited locus is solely defined by the repair template and long plasmid template homology directed repair also is possible with only one guide RNA [10, 48], but insertion rate is generally higher when using multiple guides [92, 112, 113]. In addition, cut sites must not be too distant from the site to be edited in order to avoid homologous recombination between the two sites [113]. Besides tagging genes at their 3’ ends, it has been shown that the 2A peptide sequence can be strategically placed at the 5’ end of the gene just before the translation start codon to enable N-terminal tagging [25]. This facilitates the co-expression of the upstream marker and the tagged gene without compromising its function and expands the versatility of the use of 2A peptide and CRISPR/Cas for gene tagging.

### Homology directed repair: precise editing with large cargoes using linear DNA with short homology arms

Studies on different animal model systems have shown that fragments with homology arms as short as 20–40 bp can faithfully integrate into the genome following a CRISPR induced DSB [114–118], most likely driven by a microhomology mediated end joining pathway (MHEJ) [35]. We are not aware of any study in non-model insects where homology regions of that size have successfully been used for genomic integration, and instead repair plasmids with long homology arms of 400–1000 bp (Fig. 1E) are often used [10, 43]. However, in *Drosophila melanogaster* integration of linear single stranded DNA (ssDNA) donors of multiple kb has been successful under the use of short (100 bp) homology arms [104]. In honeybees genomic integration of cargoes of up to 1 kb flanked by homology arms of 250 bp provided as linear dsDNA donors has been highly efficient [119], although this may have followed classical HDR rather than a MHEJ mechanism (Fig. 1D). Experimentally the production of linear repair templates with short homology regions is quicker and easier than the synthesis of repair plasmids with homology arms of up to 1 kb per side. Given that the use of relatively short homology regions has been highly efficient in some species [104, 116, 119] it may constitute a preferred approach to many genome editing experiments. However, further testing in additional species is necessary, also with respect to the cargo size that can be successfully integrated by this approach. In sea urchins the integration efficiency of an app. 700 bp long linear donor has been greatly increased by chemical modifications of the open ends of the donor template to avoid DNA concatenation [116].

### Screening and stock building after CRISPR-Cas9 induced transgenesis

The principles of building up a homozygous stock from a CRISPR-Cas9 based transgenesis experiment are similar as described above (section Editing the germline and establishment of a mutant stock, Fig. 4D–F). Most transgenesis constructs include a dominant visible marker gene such as GFP or DsRed*,* or genes such as *white* and *vermillion* that convert strains with an eye mutant phenotype back to the wildtype phenotype [76, 120–122]. As these markers are normally visible as soon as one allele is present [107, 120–122], they are very useful for identifying heterozygous carriers of the transgenes (Fig. 4E–F) but in many cases they may not allow differentiating between homo- and heterozygous animals. Therefore, additional molecular testing by PCR amplification of the target site is necessary [105]. Alternatively, a test cross can be performed to identify homozygous animals in which individuals displaying the dominant transformation marker are individually crossed to their wild type counterparts. All progeny of homozygous animals should show expression of the marker gene whereas 50% of the offspring of heterozygous individual display the transformation marker and 50% are wild type. PCR amplification and sequencing of the transformed locus is easiest with gDNA from homozygous individual as the presence of the wild type allele may complicate some PCR approaches.

## Conclusions and future directions

The feasibility of performing CRISPR experiments has been shown in numerous insects. Whereas the application of CRISPR-based techniques is at an advanced level in *Drosophila* species, in the important disease vector mosquito species, as well as in the model beetle *Tribolium castaneum,* experiments on other species are so far often limited to proof of principle experiments such as the targeting of eye pigmentation genes (e.g. [20–22, 29]). The targeting of genes with unknown biological function might in some cases be complicated by the lack of a visible phenotype by which mutant allele carriers can be identified or a high mortality of homozygous mutant allele carriers. The streamlining of molecular screening methods (Fig. 5) as well as the more widespread use of visible markers to be inserted along with an intended mutation can be ways to overcome these challenges (see section Using CRISPR-Cas9 to create gene knock-outs).

CRISPR-based transgenesis can also be limited by a low efficiency of HDR in some species in which it has been tried. In case of the beetle *Tribolium castaneum* HDR mediated insertion of transgenes has been successful at a relatively high efficiency for transgene replacement (6 % of G_0_-larvae produced offspring with the desired editing outcome [43 ]). However, HDR editing of endogenous loci only worked at a low efficiency in the hands of some authors (0.54-0.64 % gene edited founders per screened G_0_ animals [123]), whereas other attempts have failed [124]. In our own HDR gene tagging experiments in *Tribolium* we achieved efficiencies of 0.25-0.35 % for precise *in frame* integration of a marker gene (VSH/LZ *unpublished*). On the other hand, HDR was very efficient in honeybees with up to 50% insertion of *gfp* even in G_0_-animals [25, 26]/(M. Beye *pers. communication*). To find ways to overcome the difficulties of applying HDR in new species it is recommended, besides the identification of sgRNAs with high efficiencies, to also test different concentrations of sgRNAs, Cas9 protein/plasmid and repair template. Additionally, it is important to understand the competition between different repair mechanisms (see [35]) and to find ways to shift the ratio towards HDR. An improved nuclear targeting could be achieved in *Drosophila* by using plasmids in which the HDR repair template is provided together with a sgRNA cassette, such as the Janelia Atalanta plasmids [125].

In summary, the CRISPR-Cas9 system has transformed the field of functional genetics in non-model insects, including in agricultural pests and disease vectors, and many basic biology questions can now be addressed in diverse species using this technique. It has also greatly enhanced the versatility of species specific genetic pest control strategies with great potential for disease eradication and crop protection.

## Data Availability

Not  applicable.

## Abbreviations

Bp: Base pairs
Cas: CRISPR associated protein
CRISPR: Clustered regularly interspaced palindromic repeats
crRNA: CRISPR derived RNA
DSB: Double strand break
DiPa CRISPR: Direct parental CRISPR
dsDNA: Double stranded DNA
GFP: Green fluorescent protein
gDNA: Genomic DNA
gRNA: Guide RNA
HA: Homology arm
HDR: Homology directed repair
HR: Homologous recombination
HRMA: High resolution melt analysis
Kb: Kilobase
NHEJ: Non-homologous end joining
NLS: Nuclear localization signal
Nt: Nucleotide
ORF: Open reading frame
PAM: Protospacer adjacent motif
PCR: Polymerase chain reaction
pgSIT: Precision guided sterile insect technique
ReMot: Receptor mediated uptake of cargo
sgRNA: Single guide RNA
SIT: Sterile insect technique
ssDNA: Single stranded DNA
ssODN: Single stranded oligodeoxynucleotides
ssRNA: Single stranded RNA
tracrRNA: Transacting CRISPR derived RNA
tRNA: Transfer RNA
UTR: Untranslated region
